# Social Vulnerability and Structural Determinants of Child and Adolescent Well-Being in Europe: A Longitudinal Cross-National Analysis Using Eurostat Data (2017–2023)

**DOI:** 10.3390/healthcare14142070

**Published:** 2026-07-10

**Authors:** David Pérez-Jorge, Miriam Catalina González-Afonso, Iris Alexia Hernández-González, María Carmen Martínez-Murciano

**Affiliations:** Department of Didactics and Educational Research, Faculty of Education, University of La Laguna, 38204 San Cristobal de La Laguna, Spain; mcglez@ull.edu.es (M.C.G.-A.); ihernang@ull.edu.es (I.A.H.-G.)

**Keywords:** child social vulnerability, child poverty, social protection, inequality, social determinants of health, Europe, Eurostat

## Abstract

**Background/Objectives**: Child social vulnerability is a multidimensional phenomenon influenced by poverty, material deprivation, inequality, and institutional protection. This study analysed child and adolescent social vulnerability in Europe between 2017 and 2023 and developed a Child Social Vulnerability Index (CSVI). **Methods**: A longitudinal ecological study was conducted using Eurostat country-year data from European countries. Indicators included severe child poverty, severe material deprivation, income inequality (Gini index), preventive healthcare expenditure, and social protection expenditure. Descriptive, longitudinal, principal component, and cluster analyses were performed. **Results**: The findings revealed substantial differences in child social vulnerability across European countries. Poverty, material deprivation, and inequality were strongly associated, supporting the multidimensional nature of vulnerability. Principal Component Analysis supported the internal structure of the child Social Vulnerability Index (CSVI), identifying two components that explained 76.35% of the variance. The CSVI showed a very high correlation with the first principal component (r = 0.97, *p* < 0.001) confirming its structural consistency. Longitudinal analyses revealed a significant decline in child social vulnerability between 2017 and 2023 with an additional reduction during the COVID-19/post-COVID period. Cluster analysis identified three distinct European vulnerability profiles based on the CSVI and institutional protection dimension. **Conclusions**: Child social vulnerability in Europe is shaped by the interaction of socioeconomic inequalities and institutional protection mechanisms. The CSVI provides a useful tool for monitoring vulnerability and informing policies to reduce inequalities and promote child and adolescent well-being.

## 1. Introduction

Child and adolescent health and well-being are currently recognised as international priorities within the fields of public health, social protection, and sustainable development. Numerous international organisations have highlighted that living conditions during childhood exert a decisive influence on health, cognitive development, academic achievement, emotional well-being, and future opportunities for social and economic participation. Consequently, reducing the inequalities affecting children and adolescents has become one of the major challenges facing contemporary public policies.

Although child well-being has traditionally been examined from psychological, educational, or clinical perspectives, increasing attention has been devoted in recent decades to the study of the structural social determinants shaping child development trajectories. Factors such as poverty, material deprivation, economic inequality, family structure, and the institutional capacity for social protection create contexts of vulnerability that may restrict developmental opportunities and increase exposure to social and health-related risks throughout the life course.

Available scientific evidence indicates that child vulnerability represents a complex and multidimensional phenomenon that cannot be explained exclusively through income-related or economic poverty indicators [1]. Rather, experiences of vulnerability typically emerge from the interaction of multiple social, economic, and institutional factors that accumulate over time and generate persistent inequalities in the living conditions of minors. From this perspective, it is necessary to advance towards approaches capable of simultaneously integrating different dimensions of risk and protection in order to achieve a more comprehensive understanding of the inequalities affecting childhood.

Across Europe, substantial structural differences persist between countries regarding levels of child poverty, material deprivation, economic inequality, healthcare expenditure, social protection, and institutional support for families. These disparities reflect not only different levels of economic development but also variations in the capacity of welfare systems to protect children from situations of social vulnerability. Comparative analyses of these indicators enable the identification of inequality patterns, improve understanding of the evolution of the structural determinants of child vulnerability, and facilitate the evaluation of the role of public policies in reducing social disparities among European countries.

Furthermore, the period from 2017 to 2023 is particularly relevant for the study of child vulnerability in Europe, as it encompasses both the years preceding the COVID-19 pandemic and the subsequent phases of health crisis and recovery. The pandemic represented an event of profound social and economic impact that altered the living conditions of millions of families, increased the risks of social exclusion, and tested the capacity of public systems to respond effectively to emergency situations. At the same time, it prompted the implementation of significant social protection measures, economic support programmes, and healthcare investments that may have contributed to mitigating some of its effects on young people.

Despite the growing interest in the study of child poverty and the well-being of children and adolescents in Europe, much of the available evidence has focused on isolated indicators or cross-sectional analyses conducted at specific points in time. Studies integrating multiple dimensions of social vulnerability through comparable cross-country indicators and examining their longitudinal evolution throughout the pre- and post-COVID period remain less common. Likewise, although several multidimensional frameworks have been developed to assess child well-being, the availability of tools specifically designed to measure child social vulnerability from a structural and institutional perspective remains limited.

Within this context, the present study seeks to provide empirical evidence on the social determinants associated with child and adolescent vulnerability in Europe through the longitudinal analysis of comparable Eurostat indicators. Furthermore, it proposes the development of a CSVI integrating multiple dimensions related to poverty, material deprivation, economic inequality, and the protective capacity of public systems. In doing so, the study aims to contribute to a more comprehensive understanding of the inequalities affecting European children and to provide useful evidence for the design of public policies aimed at prevention, social protection, and the promotion of child and adolescent well-being.

### 1.1. Child Social Vulnerability as a Multidimensional Phenomenon

Difficulties experienced during early childhood tend to cluster or co-occur and, although there is considerable evidence that poverty constitutes a major risk indicator [2], child social vulnerability cannot be understood solely through this factor nor analysed at a single point in time. An approach focused exclusively on income is necessary for identifying situations of economic disadvantage; however, it provides only a partial view of a complex phenomenon.

During childhood and adolescence, conditions of vulnerability tend to emerge as cumulative processes in which low income, material deprivation, family insecurity, reduced access to educational resources, pressures on caregiving, and exposure to less protective environments converge with varying degrees of intensity [3,4]. These dimensions do not always occur simultaneously, nor do they affect all children and adolescents in the same way; however, when they accumulate, they cease to be isolated circumstances and become structural conditions that shape opportunities for development and well-being.

Several studies have shown that living conditions during early childhood can significantly influence health, learning, behaviour, and well-being throughout childhood and adolescence, especially when exposure to adversity is prolonged over time and is not accompanied by adequate family, school, or community support [4,5,6,7,8,9]. These early inequalities may extend into adulthood, affecting educational, occupational, and social advancement [10,11,12]. At the same time, evidence indicates that early investments in health, education, and child development can reduce these inequalities and generate sustained benefits throughout the life course [13].

### 1.2. Poverty, Material Deprivation and Inequality as Structural Determinants

The scientific literature has extensively documented the relationship between household socioeconomic circumstances and child well-being [14,15]. On average, children growing up in low-income households exhibit poorer health indicators and achieve less favourable outcomes in cognitive, social, and behavioural development assessments than those from higher-income households. These differences subsequently translate into poorer educational outcomes, lower self-esteem during adolescence, and a greater likelihood of developing disruptive behaviours [16].

Several authors have highlighted that family income shapes children’s opportunities through multiple mechanisms related to material deprivation, inequality of opportunity, and the institutional capacity for protection [17,18,19]. In this regard, understanding the advantages related to contexts characterised by higher levels of investment in child protection policies is essential for identifying effective strategies to reduce social inequalities.

Longitudinal evidence also demonstrates that poverty tends to coexist with other forms of adversity. Adjei et al. [2], using data from the UK Millennium Cohort Study involving 11,564 children followed up to the age of 14 years, identified six distinct trajectories related to poverty and family adversity: low poverty, low family adversity, persistent substance use, persistent domestic violence, persistent poor parental mental health, persistent poverty, and a combination of persistent poverty and persistent poor parental mental health. The findings showed that young people exposed to persistent trajectories of poverty and adversity experienced poorer socio-emotional, behavioural, and cognitive outcomes during adolescence [20,21]. Furthermore, the combination of persistent poverty and poor parental mental health was associated with higher levels of cognitive impairment, drug experimentation, and obesity at 14 years of age. Similar findings were reported by [3], who identified socioeconomic factors, including deprivation coupled with employment and education, as some of the most relevant predictors of emotional and behavioural problems.

Socioeconomic disadvantage has also been linked to increased vulnerability to multiple diseases during adolescence and adulthood [22], to the extent that some authors have described social inequalities as “the causes of the causes” of numerous health problems. Although the causal pathways linking income and health are complex, families with greater economic resources generally benefit from better access to healthcare services, high-quality nutrition, and safer living environments. Nevertheless, behavioural and cultural factors tied to socioeconomic status, such as sleep habits, preventive practices, and safety-related behaviours, also contribute to explaining part of the observed inequalities [23].

### 1.3. Social Protection, Sustainable Development and Child Wellbeing

Growing concern about inequalities affecting children has gained particular prominence since the adoption of the Sustainable Development Goals (SDGs) in 2015. Reducing child poverty has become one of the fundamental pillars of the international sustainable development agenda [24], highlighting the need for coordinated actions across areas such as health, education, housing, nutrition, social protection, environmental sustainability, and the regulation of commercial environments that influence child well-being [13,25].

Within this context, the living conditions of minors in the European Union have received greater attention. The pioneering study by Bradshaw et al. [26], based on the development of a multidimensional child well-being index, demonstrated that the differences observed among European countries could not be explained solely by levels of national wealth. Subsequently, Bradshaw and Richardson [27] reinforced this perspective by emphasising the importance of material, educational, health-related, relational, subjective, and safety dimensions in understanding child well-being. These contributions have helped consolidate the view that processes of child vulnerability and well-being should be analysed through multidimensional approaches that simultaneously incorporate economic, social, and institutional factors.

### 1.4. COVID-19, Digital Exclusion and New Forms of Vulnerability

The transformations brought about by the COVID-19 pandemic revealed new forms of child social vulnerability. The interruption of face-to-face educational activities generated significant digital inequalities, digital divides, and situations of technological exclusion that hindered the continuity of learning processes, mainly among children and adolescents from households with fewer economic resources [28,29,30].

Several studies have shown that educational opportunities during periods of lockdown were strongly conditioned by families’ economic and cultural capital [31,32]. Similarly, differences in school closure policies and economic support measures implemented across European countries reflected varying levels of priority assigned to children during the public health crisis [33,34]. These circumstances contributed to exacerbating pre-existing inequalities and highlighted the crucial role of public social protection systems in mitigating the effects of crises.

### 1.5. Single-Parent Families and Differential Vulnerability

Among the factors linked to child vulnerability, single-parenthood has received substantial attention in the international literature. Several studies have shown that single-parent households face higher risks of child poverty and material deprivation compared with other family structures [35,36,37,38,39]. However, the evidence also indicates that single-parenthood is not regarded as a direct or automatic cause of poverty, but rather a condition whose influence depends on multiple contextual factors.

Single-parent households are highly heterogeneous, and their living conditions are shaped by variables such as educational attainment, employment status, work intensity, the availability of family and community support, housing conditions, and the resources available for childcare [38,40,41,42,43,44]. Consequently, single-parenthood should be understood as a differential dimension of vulnerability whose significance depends on its interaction with other socioeconomic and institutional factors.

### 1.6. The Need for Multidimensional Indicators of Child Vulnerability

The multidimensional nature of child vulnerability has stimulated the development of various approaches aimed at synthesising conditions of well-being and deprivation through composite indicators [45,46,47]. Within the European context, the child well-being index developed by Bradshaw et al. [26] has served as a key reference for the comparative analysis of the living conditions of children and adolescents.

However, although these approaches have significantly advanced the understanding of child well-being, there remains a need to develop instruments specifically designed to measure child social vulnerability from a structural perspective. Recent literature continues to emphasise the importance of simultaneously incorporating dimensions related to poverty, material deprivation, economic inequality, and the protective capacity of public systems [48].

Consequently, it is pertinent to develop a CSVI that synthesises the level of child social vulnerability in each country and year based on comparable Eurostat indicators. Unlike indices focused on general child well-being, the CSVI is specifically intended to assess the accumulation of structural risk factors associated with severe child poverty, severe material deprivation, economic inequality, and the institutional capacity for social and healthcare protection.

The absence of synthetic tools integrating these dimensions from a longitudinal and comparative perspective within the European context, in particular during the pre- and post-COVID periods, justifies the present study and underpins the proposed objectives.

The general objective of this study is to analyse child and adolescent social vulnerability in Europe between 2017 and 2023 using comparable structural indicators from Eurostat.

The specific objectives are:

1. To describe temporal trends in child vulnerability indicators across Europe between 2017 and 2023.
2. To compare levels of child vulnerability across European countries.
3. To analyse the relationships between child poverty, material deprivation, and economic inequality.
4. To examine the association between social protection, preventive expenditure, and child vulnerability.
5. To develop a CSVI.
6. To analyse the effect of the COVID-19 and post-COVID periods on child vulnerability.
7. To identify profiles of European countries according to their levels of social vulnerability and institutional protection.

## 2. Materials and Methods

### 2.1. Study Design

A longitudinal ecological study was conducted to analyse the evolution of the structural determinants in conjunction with child and adolescent social vulnerability in Europe during the period 2017–2023.

The unit of analysis consisted of the country–year observations available for each of the selected indicators. This design enabled the simultaneous examination of cross-country differences and temporal changes occurring throughout the study period, including the years preceding the COVID-19 pandemic and the subsequent post-pandemic stage.

Descriptive, correlational, longitudinal, and multivariate analyses were performed to characterise levels of child vulnerability, examine the relationships among the different structural indicators, and develop a CSVI based on socioeconomic and institutional variables comparable across European countries.

### 2.2. Data Sources

The data were obtained from Eurostat, the statistical office of the European Union, which provides harmonised, standardised, and comparable information on socioeconomic conditions, inequality, health, and social protection across European countries.

Annual indicators corresponding to the period 2017–2023 were collected for European countries with sufficient data availability. The use of Eurostat ensures the methodological comparability of indicators across countries and represents one of the principal data sources for comparative studies on social inequality and child well-being in Europe.

### 2.3. Variables and Indicators

The study included structural indicators related to child poverty, material deprivation, economic inequality, and institutional protection.

The socioeconomic variables incorporated were:

severe child poverty (40% of the equivalised median income threshold) among children aged 6–11 years;

severe child poverty (40% of the equivalised median income threshold) among adolescents aged 12–17 years;

- severe material deprivation among children aged 6–11 years;
- severe material deprivation among adolescents aged 12–17 years;
- Gini index for the population under 18 years of age.

The following institutional indicators were included:

- preventive healthcare expenditure per capita (PPS);
- social protection expenditure per capita (PPS).

Both indicators were expressed in Purchasing Power Standards (PPS), thereby improving comparability across countries by adjusting for differences in purchasing power.

In addition, indicators related to single-parent households were explored as a potential dimension associated with child vulnerability. However, due to the high proportion of missing values and limited longitudinal availability, these variables were excluded from the main construction of the CSVI and were used only in complementary descriptive analyses.

### 2.4. Data Management and Sample Analysis

The initial database comprised 301 country–year observations corresponding to European countries during the period 2017–2023.

The analysis of missing values revealed a heterogeneous distribution across the variables included in the study. Severe child poverty and severe material deprivation each presented 49 missing values (16.28%), while the Gini index recorded 57 missing observations (18.94%). Among the variables used to construct the CSVI, preventive healthcare expenditure showed the highest proportion of missing data (87 observations; 28.90%), followed by social protection expenditure (65 observations; 21.59%).

Missing information was concentrated primarily in Bosnia and Herzegovina, Liechtenstein, and Kosovo, as well as in certain European territorial aggregations. Furthermore, the proportion of missing data increased moderately in the most recent years, chiefly for indicators related to poverty and inequality.

Variables in relation to single-parenthood exhibited particularly high levels of missing information (34.55–95.35%). Although an aggregated indicator of single-parent households was constructed, only 119 valid observations were available (39.53% of the total sample). Consequently, this variable was excluded from the main construction of the index.

Finally, the CSVI was calculated using 198 complete observations (65.78% of the original database) through listwise deletion, thereby ensuring the structural comparability of subsequent multivariate analyses.

## 3. Results

### 3.1. Descriptive Analysis and Trends over Time in Child Social Vulnerability (2017–2023)

#### 3.1.1. General Descriptive Statistics

The results indicated marked heterogeneity across European countries in the main indicators of child social vulnerability. Severe child poverty (40% of the equivalised median income threshold) showed a mean prevalence of 10.00% among children aged 6–11 years and 11.54% among adolescents aged 12–17 years. Severe material deprivation displayed similar values across age groups, with mean rates of 10.20% and 9.99%, respectively.

The Gini index among individuals under 18 years of age reached a mean value of 29.84 points (SD = 5.52), ranging from 20.60 to 46.80, reflecting substantial differences in economic inequality across European countries (Table 1). Moreover, preventive healthcare expenditure averaged 103.44 PPS per capita (SD = 81.03), whereas social protection expenditure reached 115.84 PPS per capita (SD = 143.87), indicating considerable variability in the institutional capacity for social and healthcare protection. 

#### 3.1.2. Differences Between European Countries

**Figure 1:**
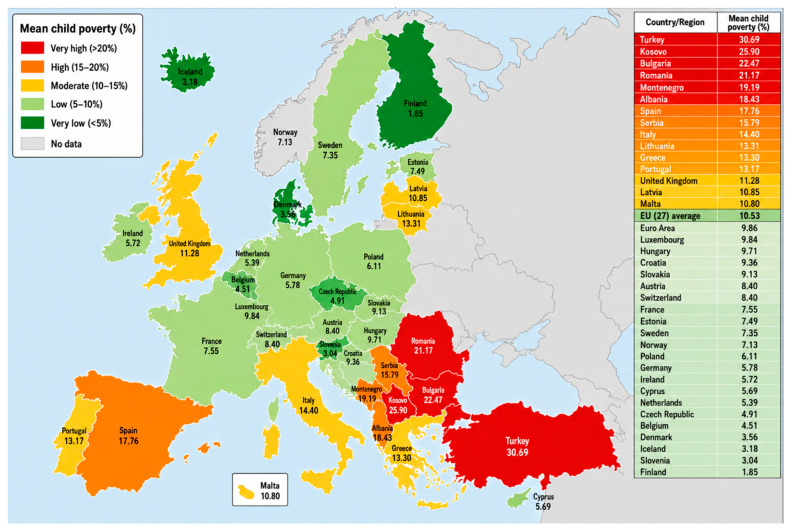
Geographical distribution of average child poverty in Europe (2017–2023).

Marked territorial inequalities were observed in the levels of child social vulnerability across European countries (Figure 1). The highest levels of child poverty were concentrated primarily in Southern and Eastern European countries, primarily Türkiye (30.54% among children aged 6–11 years and 30.84% among adolescents aged 12–17 years), followed by Kosovo, Bulgaria, and Romania. Romania also exhibited high levels of severe material deprivation (29.51–30.54%) and economic inequality (Gini = 36.43).

In contrast, Finland, Iceland, Slovenia, and Denmark recorded the lowest levels of child vulnerability. Finland reported only 1.80% child poverty among children aged 6–11 years and 1.89% among adolescents, together with minimal levels of severe material deprivation.

Regarding institutional protection, Germany showed one of the highest levels of preventive healthcare expenditure (243.74 PPS per capita), while Germany and Luxembourg recorded the highest investments in social protection (622.93 and 546.55 PPS per capita, respectively).

Overall, the findings reveal a consistent geographical pattern of child social vulnerability across Europe, distinguished by higher levels of poverty and inequality in countries with lower institutional capacity for social protection and prevention.

#### 3.1.3. Temporal Trends

The longitudinal descriptive analyses revealed a moderate reduction in child social vulnerability across Europe between 2017 and 2023, as shown in Table 2. Mean child poverty declined from 11.54 in 2017 to 10.31 in 2023, while severe material deprivation decreased from 12.28 to 9.19 over the same period. Similarly, the Gini index showed a slight reduction, declining from 30.66 in 2017 to 29.23 in 2023.

Institutional indicators exhibited an upward trend, particularly during the COVID-19 pandemic period. Preventive healthcare expenditure increased from 66.72 PPS per capita in 2017 to a peak of 179.60 PPS in 2021, before subsequently declining to 97.78 PPS in 2023. Social protection expenditure showed a progressive increase throughout the study period, rising from 110.03 PPS per capita in 2017 to 131.29 PPS in 2023.

Taken together, these findings indicate that increases in preventive healthcare expenditure and social protection expenditure coincided temporally with a moderate reduction in child social vulnerability across Europe.

### 3.2. Construction and Validation of the CSVI

The CSVI was constructed using five standardised structural indicators transformed into z-scores: child poverty, severe material deprivation, economic inequality (Gini index), inverted preventive healthcare expenditure, and inverted social protection expenditure. The institutional variables were reverse-coded so that higher index scores would represent higher levels of child social vulnerability.

Before constructing the CSVI, the age-specific poverty and severe material deprivation indicators were aggregated to obtain a single measure for each domain. Specifically, the indicators for children aged 6–11 years and adolescents aged 12–17 years were combined by calculating their arithmetic mean, giving equal weight to both age groups. This approach preserved information from both developmental stages while simplifying the structure of the composite index. The aggregated indicators were subsequently standardised together with the remaining variables and incorporated into the CSVI.

Equal weighting (0.20 per indicator) was adopted following the recommendations of the OECD Handbook on Constructing Composite Indicators [49], which identifies equal weighting as the most commonly used and appropriate approach when there is insufficient theoretical or empirical evidence to justify differential weights among indicators. This strategy enhances transparency, parsimony, and interpretability while avoiding arbitrary weighting decisions. Principal Component Analysis (PCA) was subsequently used to assess the structural validity of the index rather than to derive indicator weights.

The general formulation of the index was as follows:

$$CSVI=\frac{Z_{child\;poverty}+Z_{severe\;material\;deprivation}+Z_{Gini\;index}+Z_{inverted\;preventive\;healthcare\;expenditure}+Z_{inverted\;social\;protection\;expenditure}}{5}$$

Note. The Child Social Vulnerability Index (CSVI) was calculated as the arithmetic mean of five standardised indicators (z-scores): child poverty, severe material deprivation, economic inequality (Gini Index), inverted preventive healthcare expenditure, and inverted social protection expenditure. The institutional indicators were reverse-coded by multiplying their standardised values by −1 so that higher scores consistently reflected higher levels of child social vulnerability. Standardisation was applied to ensure comparability among variables measured on different scales. Consequently, higher CSVI values indicate greater child social vulnerability, whereas lower values reflect more favourable socioeconomic and institutional conditions.

The correlation analyses identified strong and consistent associations among the main socioeconomic indicators of vulnerability. The highest correlation was observed between child poverty and economic inequality (r = 0.848), followed by the association between child poverty and severe material deprivation (r = 0.693). Likewise, severe material deprivation showed a moderate-to-strong positive correlation with the Gini index (r = 0.543), reflecting the close relationship between economic inequality and adverse material conditions during childhood (Table 3).

In contrast, the institutional variables exhibited inverse associations with vulnerability indicators. Because preventive healthcare expenditure was reverse-coded, the positive correlations indicate that lower levels of preventive investment were associated with higher levels of child poverty (r = 0.276) and severe material deprivation (r = 0.342). Similarly, because social protection expenditure was also reverse-coded, the positive correlations observed with child poverty (r = 0.099), severe material deprivation (r = 0.176), and inverted preventive healthcare expenditure (r = 0.341) indicate that lower levels of institutional protection tended to coexist with higher levels of social vulnerability. These findings support the internal coherence of the composite index and reinforce the interpretation of institutional investment as a protective factor against child social vulnerability.

#### Principal Component Analysis

The Principal Component Analysis (PCA) applied to the five CSVI variables showed a robust and coherent factorial structure. The first principal component (PC1) had an eigenvalue of 2.571 and explained 51.42% of the total variance, whereas the second component (PC2) had an eigenvalue of 1.247 and accounted for an additional 24.93% of the variance. Together, these two components explained 76.35% of the cumulative variance.

**Figure 2:**
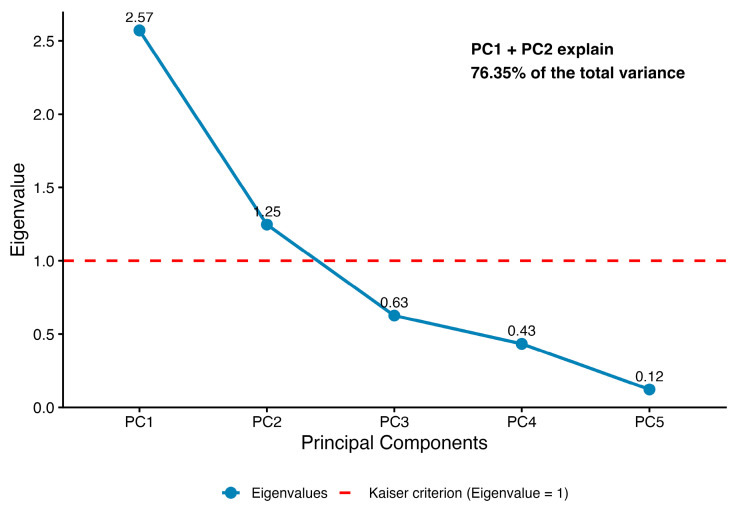
Scree plot of the principal component analysis.

The remaining components presented eigenvalues below 1 and substantially lower contributions and were therefore not retained according to the Kaiser criterion. As shown in Figure 2, the scree plot visually confirmed the retention of two principal components as the optimal factorial solution.

Table 4 presents the eigenvalues and the percentage of variance explained by each principal component. PCA was performed as a validation procedure to examine the latent structure underlying the CSVI rather than as a weighting technique. This approach follows OECD recommendations for assessing the internal coherence of composite indicators constructed using equal weighting.

As shown in Table 5, the factor loadings indicated that PC1 represented a global dimension of structural vulnerability, displaying high positive loadings for child poverty (0.578), economic inequality (0.525), and severe material deprivation (0.521), together with moderate contributions from inverted preventive healthcare expenditure (0.309) and inverted social protection expenditure (0.153). Overall, this component primarily captured the structural burden of children’s socioeconomic vulnerability.

In contrast, PC2 reflected a dimension mainly aligned with institutional protection, with high loadings for inverted social protection expenditure (0.734) and inverted preventive healthcare expenditure (0.558). This component enabled differentiation between countries with higher levels of public investment and institutional capacity for social protection and those with lower preventive capacity.

The variables contributing most strongly to PC1 were child poverty and economic inequality, consistent with the high correlation between them (r = 0.848).

The correlation between the final CSVI and the first principal component was extremely high (r = 0.967, *p* < 0.001), confirming the structural consistency and internal validity of the constructed index.

### 3.3. Longitudinal Models

The longitudinal analyses were conducted using an unbalanced panel comprising 31 European countries and 198 country–year observations covering the period 2017–2023.

Both fixed-effects and random-effects models produced very similar estimates. Because the Hausman test was not significant (χ^2^ = 0.435; *p* = 0.80), the random-effects model revealed a significant decrease in the CSVI over time (β = −0.028; robust SE = 0.013; *p* = 0.030), indicating a progressive reduction in structural child vulnerability throughout the study period (Table 6). Furthermore, the COVID-19/post-COVID period (2020–2023) was associated with significantly lower CSVI values than the pre-pandemic period (2017–2019) (β = −0.155; robust SE = 0.053; *p* < 0.01). This reduction coincided temporally with the observed increase in preventive investment and social protection expenditure during the pandemic. The model explained 36.6% of the within-country variability (R^2^ = 0.243), indicating a moderate explanatory capacity.

**Figure 3:**
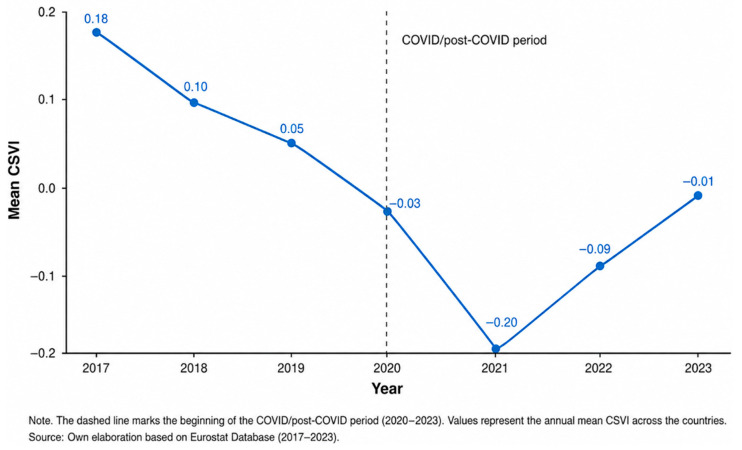
Temporal Evolution of the CSVI in Europe (2017–2023).

Figure 3 shows a progressive decline in the mean CSVI between 2017 and 2021, reaching its lowest point in 2021, followed by a recovery in 2022 and 2023. This descriptive pattern is consistent with the longitudinal random-effects model, which identified a negative temporal trend and a significant reduction in the CSVI during the COVID-19/post-COVID period. Given that preventive healthcare spending and social protection are components of the CSVI, these institutional changes should be interpreted as part of the index’s structure, rather than as external causal explanations.

### 3.4. Cluster Analysis and European Vulnerability Profiles

The three-cluster solution was selected because it provided the best balance between statistical separation and substantive interpretability. Cluster validity was assessed using the average silhouette coefficient, which reached 0.494, indicating a moderate clustering structure with acceptable separation between clusters and supporting the retention of the three-cluster solution.

The cluster analysis was conducted using the k-means method on the aggregated country-level dataset (31 countries), obtained by averaging the country-year observations included in the longitudinal analyses. The analysis was based on the national mean CSVI scores and the institutional protection dimension (Table 7).

The first cluster is a Dual Vulnerability and Structural Risk that comprises countries (e.g., Greece, Spain, Romania, Serbia) that present the most critical profile. It is characterised by a negative convergence between a high child social vulnerability index (CSVI = 0.812) and a marked weakness in institutional protection mechanisms (0.572). Children not only face higher levels of poverty and material deprivation, but are also deprived of a robust public safety net in terms of preventative spending and social protection. This scenario suggests a systemic structural risk, where the absence of compensatory policies intensifies the impact of economic inequality, perpetuating cycles of vulnerability that the state is unable to mitigate effectively.

Cluster 2 is Institutional Resilience and European Convergence, and is the most representative in terms of sample size (e.g., Austria, France, Norway, Poland). It reflects the operating principles of the European Social Model. This group exhibits values centred around the regional average, with a slight tendency towards protection (CSVI = −0.270; institutional vulnerability = −0.066). It displays a profile of moderate institutional resilience, where states manage to balance socio-economic pressures through functional welfare systems. Although these countries are not immune to risks, the architecture of their public policies acts as a stabiliser that prevents child vulnerability from escalating to critical levels, placing them in a position of convergence with the European Union’s welfare standards.

**Figure 4:**
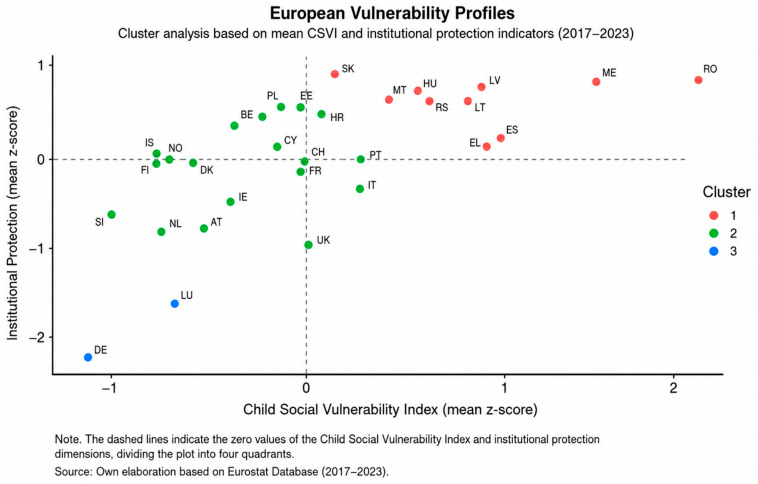
European Clusters According to Child Social Vulnerability Profiles.

Cluster 3 was identified as ‘The Institutional Protection and Advanced Well-being Paradigm’. As a highly specialised cluster (e.g., Germany, Luxembourg), it represented the most favourable profile in the sample. These countries exhibit a virtuous correlation between the lowest levels of child vulnerability (CSVI = −0.906) and unprecedented institutional investment, evidenced by an extremely low institutional vulnerability score (−2.060). This result points to ‘institutional shielding’: a configuration in which massive spending on preventive healthcare and social protection acts as a proactive shield, neutralising the social determinants of vulnerability. Children’s economic security does not depend solely on market dynamics or household income, but on high-intensity state intervention that guarantees superior standards of protection (Figure 4).

Overall, these results indicate that child social vulnerability in Europe is a multidimensional structural phenomenon that encompasses both socioeconomic disadvantage and institutional conditions related to social protection and preventive healthcare.

## 4. Discussion

This study longitudinally examined the evolution of structural determinants related to child and adolescent social vulnerability in Europe between 2017 and 2023 through the development of the CSVI, based on indicators of poverty, material deprivation, economic inequality, and institutional protection capacity. The findings provide several important contributions to both the understanding of child vulnerability and the design of public policies aimed at protecting children and adolescents.

In particular, the study helps address the scarcity of comparative longitudinal analyses specifically focused on child social vulnerability in Europe and provides empirical evidence regarding the interaction between socioeconomic factors and institutional protection mechanisms.

Beyond confirming the multidimensional nature of child vulnerability described in previous research, the present study offers a comparative and longitudinal perspective that enables the simultaneous examination of temporal changes in structural risk and protective factors across different European countries. Furthermore, it proposes a synthetic index specifically designed to measure child social vulnerability, complementing child well-being approaches developed in previous studies and providing a useful tool for monitoring child inequalities in European contexts. This contribution is particularly relevant given the limited availability of comparable composite indicators for the longitudinal analysis of child vulnerability across countries. Unlike approaches primarily focused on child well-being, the CSVI is specifically oriented towards identifying structural risk and protective factors, thereby enabling the joint analysis of socioeconomic and institutional dimensions that are often examined separately.

The evidence obtained confirm that child social vulnerability is a clearly multidimensional phenomenon. The strong associations observed among child poverty, severe material deprivation, and the Gini index demonstrate that vulnerability cannot be interpreted solely as insufficient income but rather as the convergence of multiple forms of social disadvantage. This evidence is consistent with the contributions of numerous authors who have emphasised the need for a multidimensional approach to child well-being [25,26,27,48]. As outlined in the theoretical framework of this study, economic poverty represents an important dimension, but it is not the sole condition determining unequal experiences of growth and development during childhood.

These findings reinforce the view that child vulnerability should be understood as a cumulative process of disadvantage rather than as a condition exclusively associated with monetary poverty, reinforcing approaches that integrate material, relational, and institutional dimensions to explain inequalities during childhood.

The results also reveal marked territorial heterogeneity across European countries. The highest levels of vulnerability were concentrated mainly in Southern and Eastern European countries, whereas Nordic countries and several Central European economies exhibited significantly lower levels. This pattern is consistent with previous research identifying persistent differences among European welfare regimes regarding child poverty, social protection, and redistributive capacity [50,51,52]. The observed geographical distribution suggests that child inequalities are shaped not only by national economic characteristics but also by differences in the institutional organisation of social protection systems.

The relatively favourable position observed among Nordic countries is consistent with the literature on universal welfare regimes, exhibiting high levels of social protection, extensive family support policies, sustained investment in childhood, and stronger redistributive capacities. These characteristics may contribute to reducing the cumulative exposure of children and adolescents to poverty, material deprivation, and inequality, thereby promoting more equitable developmental trajectories.

Another important finding concerns the temporal evolution of the indicators analysed. Between 2017 and 2023, a moderate reduction was observed in child poverty, severe material deprivation, and economic inequality. Although the magnitude of these changes was relatively modest, the longitudinal models highlighted a significant decline in the CSVI throughout the study period. These findings imply a gradual improvement in the structural conditions correlated with child vulnerability across Europe, although this improvement should be interpreted cautiously given the persistence of substantial cross-country disparities.

It is noteworthy the association observed between the COVID-19/post-COVID-19 period and lower CSVI levels. Instead of interpreting this finding as a positive consequence of the pandemic, the results appear to reflect the effects of the extraordinary social protection measures implemented by many European countries during the health crisis. This interpretation is consistent with the work of Daly [33] and Hale et al. [34], who argued that the intensity and orientation of public responses could significantly shape the social consequences of the pandemic. The increases observed in preventive healthcare expenditure and social protection during the period 2020–2023 indicate that institutional intervention may have played a buffering role against the potential deterioration of children’s living conditions.

Nevertheless, because preventive healthcare expenditure and social protection form part of the CSVI construction, these findings should be interpreted as structural associations consistent with a protective role of public policies rather than as direct causal evidence of their effects on child vulnerability.

Institutional capacity emerges as one of the most significant findings of the study. Both the observed correlations and the factorial structure identified through Principal Component Analysis indicate that social protection and preventive expenditure contribute notably to explaining differences in vulnerability across countries. These findings support the social determinants of health and well-being perspective proposed by Marmot [53] and Braveman and Gottlieb [22], according to which inequalities observed during childhood largely arise from structural conditions that distribute resources, opportunities, and protective mechanisms. From this perspective, child vulnerability can be understood as the manifestation of accumulated structural inequalities affecting access to material resources, educational opportunities, healthy environments, and social protection mechanisms. Consequently, reducing these inequalities requires interventions that extend beyond the healthcare sector and address the broader social determinants shaping child well-being.

The analyses also align with recent studies on the effectiveness of social transfers and family support policies. Several studies have demonstrated that the capacity to reduce child poverty depends not only on the volume of resources invested but also on the design, targeting, and orientation of public policies [54,55,56,57,58]. In this regard, the countries exhibiting the lowest levels of vulnerability were also those with the highest levels of preventive investment and institutional protection, reinforcing the importance of public policies as mechanisms capable of moderating children’s exposure to structural social risks.

From a methodological perspective, the analyses provide favourable evidence regarding the validity of the CSVI as a synthetic instrument for the comparative analysis of child vulnerability. The PCA confirmed a coherent structure in which poverty, material deprivation, and economic inequality constituted a primary dimension of structural vulnerability, while social protection and preventive expenditure defined a second dimension associated with institutional capacity. The very high correlation observed between the CSVI and the first principal component reinforces the internal consistency of the index and supports its use as a comparative tool in international longitudinal studies.

Moreover, the strong correspondence between both measures suggests that the CSVI adequately captures the latent structure of vulnerability identified in the data while maintaining a straightforward substantive interpretation that is readily applicable to monitoring, evaluation, and policy design contexts focused on childhood.

The cluster analysis also enabled the identification of distinct profiles of European countries that extend beyond simple ranking-based comparisons. The identified groups point towards the existence of relatively stable structural configurations of vulnerability and institutional protection. This finding is consistent with comparative welfare regime research emphasising the importance of analysing combinations of socioeconomic and institutional factors instead of isolated indicators [50,52].

From an applied perspective, the findings provide evidence that reducing child vulnerability requires comprehensive strategies combining poverty reduction measures, strengthened social protection systems, and sustained investment in preventive healthcare. The results also indicate that policies focused exclusively on economic growth may be insufficient unless accompanied by redistributive mechanisms capable of reducing inequalities affecting children and adolescents. In this regard, identifying differentiated vulnerability profiles may facilitate the design of interventions better tailored to the structural characteristics of each national context.

These results should be interpreted in light of several limitations. First, the study is based on aggregated country–year data; therefore, the observed associations cannot be directly extrapolated to the individual level nor interpreted as causal relationships. Furthermore, unequal data availability across countries required the exclusion of certain observations and limited the inclusion of potentially relevant variables, such as single-parenthood, which ultimately could not be incorporated into the main index construction. The CSVI relies exclusively on indicators that are homogeneously available through Eurostat and therefore does not include other important dimensions of child vulnerability identified in recent research, such as digital deprivation [28], mental health, or intra-household inequalities [59]. In addition, the unequal availability of certain indicators across countries and years may have partially reduced the representativeness of some national contexts in the longitudinal analyses.

The CSVI was constructed using an equal-weighting strategy across the selected indicators. Although this decision enhances transparency and interpretability, future studies could explore alternative weighting procedures derived from factorial techniques or multicriteria models to assess the stability of the findings.

Future research could expand the index by incorporating additional dimensions of child vulnerability, such as mental health, digital deprivation, and intra-household inequalities, as well as by developing multilevel analyses that combine structural indicators with individual-level data. It would also be particularly valuable to evaluate the sensitivity of the CSVI to different weighting strategies and to explore its applicability in other geographical and temporal contexts in order to strengthen its usefulness as a monitoring and public policy evaluation tool.

Overall, the evidences reinforce the need to address child vulnerability from a multidimensional perspective that integrates socioeconomic factors and institutional protection capacity, highlighting the strategic role of public policies in reducing inequalities affecting children and adolescents across Europe and in promoting more equitable and healthier developmental trajectories.

## 5. Conclusions

The findings of this study confirm that child social vulnerability in Europe is a multidimensional phenomenon emerging from the interaction between child poverty, material deprivation, economic inequality, and the institutional capacity for protection. The differences observed across countries demonstrate that conditions of child vulnerability depend not only on levels of economic development but also on the capacity of public systems to provide effective mechanisms of protection and support for families.

During the period 2017–2023, a moderate reduction was observed in the indicators related to child vulnerability, coinciding with an increase in social protection expenditure and preventive healthcare spending, especially during the pandemic years and the subsequent recovery period. Although the research outcomes do not permit the establishment of causal relationships, they suggest that public policies aimed at protecting children may play an important role in mitigating structural inequalities.

Furthermore, the CSVI demonstrated an adequate capacity to synthesise socioeconomic and institutional dimensions of risk and protection, enabling the identification of differentiated profiles among European countries and facilitating the comparative analysis of child vulnerability from a longitudinal perspective.

Overall, the findings reinforce the need to promote comprehensive strategies that combine the reduction in child poverty, the mitigation of socioeconomic inequalities, and the strengthening of social protection systems and preventive healthcare services. From a public health perspective, this study provides evidence of the importance of addressing the structural determinants that shape child well-being and offers a useful tool for monitoring child social vulnerability and informing the design of policies aimed at promoting more equitable and healthier developmental trajectories across Europe.

## Figures and Tables

**Table 1:** General Descriptive Statistics of the Variables Included in the CSVI.

Variable	Valid *n*	Mean	SD	Minimum	Maximum
Poverty 40% (6–11 years)	252	10.00	6.50	0.90	33.90
Poverty 40% (12–17 years)	252	11.54	7.05	0.70	32.50
Severe Material Deprivation (6–11 years)	252	10.20	9.97	0.30	55.30
Severe Material Deprivation (12–17 years)	252	9.99	9.98	0.00	52.10
Gini Index (<18 years)	244	29.84	5.52	20.60	46.80
Preventive Healthcare Expenditure (PPS per capita)	214	103.44	81.03	10.76	499.75
Social Protection Expenditure (PPS per capita)	236	115.84	143.87	0.00	755.23

**Table 2:** Temporal Trends in Structural Indicators (2017–2023).

Year	Mean Child Poverty	Mean Severe Material Deprivation	Gini Index	Preventive Expenditure (PPS per Capita)	Social Protection Expenditure (PPS per Capita)
2017	11.54	12.28	30.66	66.72	110.03
2018	11.51	10.64	30.43	68.98	112.66
2019	10.81	9.68	29.60	74.04	108.27
2020	10.34	9.63	29.64	97.05	113.63
2021	10.51	9.43	29.73	179.60	115.98
2022	10.19	9.56	29.49	139.81	119.44
2023	10.31	9.19	29.23	97.78	131.29

**Table 3:** Correlation Matrix of the CSVI Components.

Variable	Child Poverty	Severe Material Deprivation	Gini Index	Inverted Preventive Healthcare Expenditure	Inverted Social Protection Expenditure
Child Poverty	1.000	0.693	0.848	0.276	0.099
Severe Material Deprivation	0.693	1.000	0.543	0.342	0.176
Gini Index	0.848	0.543	1.000	0.182	−0.026
Inverted Preventive Healthcare Expenditure	0.276	0.342	0.182	1.000	0.341
Inverted Social Protection Expenditure	0.099	0.176	−0.026	0.341	1.000

**Table 4:** Eigenvalues and Explained Variance of the PCA.

Component	Eigenvalue	%Variance Explained	Cumulative %
PC1	2.571	51.42	51.42
PC2	1.247	24.93	76.35
PC3	0.626	12.52	88.88
PC4	0.434	8.68	97.55
PC5	0.122	2.45	100.00

**Table 5:** Factor Loadings of the Variables Included in the PCA.

Variable	PC1	PC2
Child Poverty	0.578	−0.199
Severe Material Deprivation	0.521	0.008
Gini Index	0.525	−0.332
Inverted Preventive Healthcare Expenditure	0.309	0.558
Inverted Social Protection Expenditure	0.153	0.734

**Table 6:** Random-Effects Longitudinal Model of the CSVI.

Variable	β	Robust Standard Error	*p*-Value
Time	−0.028	0.013	0.030
COVID-19/Post-COVID Period	−0.155	0.053	<0.01

**Table 7:** Cluster Analysis of European Countries According to the Child Social Vulnerability Index (CSVI) and Institutional Protection.

Cluster	CSVI	Institutional Protection
1	0.812	0.572
2	−0.27	−0.066
3	−0.906	−2.06

## Data Availability

The data presented in this study are openly available in Eurostat at https://ec.europa.eu/eurostat/databrowser/ (accessed on 15 April 2026). The study used publicly available Eurostat datasets covering child poverty, material deprivation, income inequality, preventive healthcare expenditure, and social protection expenditure for the period 2017–2023.

## Abbreviations

The following abbreviations are used in this manuscript:

COVID-19	Coronavirus disease 2019
CSVI	Child Social Vulnerability Index
PCA	The Principal Component Analysis
SDGs	Sustainable Development Goals
EU	European Union
